# Severe Hypoglycemia Accompanied with Thyroid Crisis

**DOI:** 10.1155/2012/168565

**Published:** 2012-11-04

**Authors:** Yuki Nakatani, Tsuyoshi Monden, Minoru Sato, Nozomi Domeki, Mihoko Matsumura, Nobuyuki Banba, Takaaki Nakamoto

**Affiliations:** ^1^Department of Endocrinology and Metabolism, Dokkyo Medical University School of Medicine, 880 Kitakobayashi, Mibu, Tochigi 321-0293, Japan; ^2^Department of Diabetes & Endocrinology, Dokkyo Medical University Nikko Medical Center, 632 Takatoku, Nikko-shi, Tochigi 321-2593, Japan; ^3^Monden Clinic, 115 Tenjincho, Takasaki-shi, Gunma 370-0061, Japan; ^4^Department of Cardiology, Dokkyo Medical University Nikko Medical Center, 632 Takatoku, Nikko-shi, Tochigi 321-2593, Japan

## Abstract

We report a 32-year-old Japanese women with severe hypoglycemia accompanied with thyroid crisis. She complained of dyspnea, general fatigue, and leg edema. She was diagnosed with hyperthyroidism with congestive heart failure and liver dysfunction. Soon after admission, sudden cardiopulmonary arrest occurred. She was then transferred to the intensive care unit. Her serum glucose level was 7 mg/dl. Intravenous glucose, hydrocortisone, diuretics, and continuous hemodiafiltration (CHDF) saved her. We considered that hypoglycemia occurred due to heart failure and liver dysfunction due to thyroid crisis.

## 1. Introduction

Hypoglycemia occurs with diabetes treatment, anorexia nervosa, liver disease, and adrenal insufficiency. Hyperthyroidism usually induces impaired glucose tolerance. However, hyperthyroidism with congestive heart failure or liver dysfunction is considered to induce hypoglycemia, but this is very rare. We describe here a case of thyroid crisis accompanied with severe hypoglycemia. Although the patient's heart arrested, combined and intensive treatment saved her. 

## 2. Case Report

A 32-year-old Japanese woman was admitted to our hospital in January because of dyspnea, general fatigue, and legs edema. She had been well until one month earlier and had not been diagnosed with hyperthyroidism. There was no family history of thyroid disease.

 On admission, she was 157 cm tall and weighed 68.4 kg. Her body temperature was 36.9°C, blood pressure was 120/80 mmHg, and pulse rate was 132/min with irregularities. She was alert and oriented (Glasgow Coma Scale 15/15). Exophthalmos was not observed, but a diffuse enlarged thyroid was detected. Conjunctiva was not anemic, but was slightly icteric. Marked edema was found in both legs. Cardiac symptoms were recognized as class four of the New York Heart Association classification.

 Laboratory data indicated hyperthyroidism (free T3: >30.0 pg/mL, free T4: >6.0 ng/dL). The anti-TSH receptor antibody was elevated to 23.6 IU/L (Table 1). Electrocardiogram showed atrial fibrillation and chest X-ray showed cardiomegaly (CTR = 65.2%) and effusion. Based on these findings, the patient was diagnosed with Graves' disease complicated with severe cardiac failure. Her condition was recognized as thyroid crisis, as described by Burch and Wartofsky [1] and the Japanese Thyroid Association [2]. She was started on thiamazole, propranolol, and furosemide. In addition, we did not use steroid and iodine. Because her consciousness was clear,we thought her thyroid crisis was not severe. Her glucose level was relatively low (57 mg/dL) before meals, and she ate food at dinner time. On the first night in the hospital, she suddenly lost consciousness and suffered cardiopulmonary arrest. Immediate cardiopulmonary rescue recovered her condition. She was then transferred to the intensive care unit. Hypoglycemia (7 mg/dL), severe right-side heart failure, and liver dysfunction by liver congestion were revealed. As her condition was multiple organ failure caused by thyroid crisis, intravenous glucose, hydrocortisone, methimazole, and diuretics were administered. To maintain circulation status, continuous hemodiafiltration was performed for 7 days. As her condition improved and laboratory data reached normal ranges, she was discharged after 74 days in the hospital.

## 3. Discussion

 This case was determined as a definite case of thyroid storm by the criteria for thyroid storm described by Burch and Wartofsky (score, 85/140) [1]. In addition, the Japanese Thyroid Association has recently established the diagnostic criteria for thyroid crisis [2]. By these criteria, thyroid crisis has been diagnosed on the basis of the following five symptoms: (1) central nervous system manifestations; (2) fever (38°C or higher); (3) tachycardia (130 beats/min or faster); (4) congestive heart failure; (5) gastrointestinal manifestations. Definite cases are diagnosed by the following criteria: (1) central nervous system manifestations, plus one or more other symptoms, or (2) three or more of the manifestations other than central nervous system manifestations. On the basis of the criteria, this patient was diagnosed as a definitive case of thyroid crisis (increased serum thyroxine, central nerve symptoms (coma), tachycardia (132 beats/min), congestive heart failure, and liver dysfunction with icterus).

 This case was very rare because of hypoglycemia. In the English literature, there were three case reports of hypoglycemia accompanied by hyperthyroidism. The first case was caused by anorexia [3], the second was caused by liver dysfunction and lactic acidosis [4], and the cause of the third was not clear [5]. In the present case, insulin autoimmune syndrome was not observed because of the low level of immune reactive insulin (2.0 *μ*U/mL) and anti-insulin antibody is negative.Anorexia nervosa was rejected because of her past history and symptoms. Moreover, adrenal insufficiency was negative (ACTH: 61.3 pg/mL, cortisol: 17.3 mg/mL). Our case showed severe congestive heart failure (EF = 53.8%, IVC = 25.9 mm, respiratory changes did not occur) and liver dysfunction with icterus. Therefore, we believe that hypoglycemia may have been caused by congestive heart failure and liver dysfunction. Although lactic acid was not determined, our case resembled the second case reported by Kobayashi et al. [4]. Congestive heart failure is associated with hypoglycemia because of decreasing insulin clearance and severe liver dysfunction, which inhibits glucose release from liver cells [6].

 This case presented here is of clinical importance. Serum glucose levels should be checked in patients with thyroid crisis, especially when accompanied with heart failure and liver dysfunction. We should pay attention to glucose levels in the course of hyperthyroidism in cases like this. Fortunately, this patient was saved from thyroid crisis by intensive care including CHDF. However, we should remember hypoglycemia as a cause of loss of consciousness to avoid delayed diagnosis or management of thyroid crisis.

## Figures and Tables

**Table 1:** Laboratory findings in the intensive care unit.

AST	102 U/L
ALT	36 U/L
T-bil	2.3 mg/dL
BUN	22 mg/dL
Cre	0.58 mg/dL
Na	141 mEq/L
K	5.6 mEq/L
Cl	105 mEq/L
	
Glucose	7 mg/dL
CRP	0.1 mg/dL
	
WBC	11100/mm^3^
Hb	9.9 g/dL
Plt	14.9 × 10^4^/mL
	
F-T_3_	>30 pg/mL
F-T_4_	>6.0 ng/dL
TSH	<0.01 *μ*IU/mL
TSAb	616%
TRAb	23.6 IU/L
	
GH	4.2 ng/mL
ACTH	61.3 pg/mL
Cortisol	17.3 mg/dL
IRI	2.0 *μ*/mL
	
pH	6.949
PO_2_	82.8 mmHg
PCO_2_	57.6 mmHg
BE	−19.5 mmol/L
HCO_3_^−^	12.3 mmol/L

AST: aspartate-aminotransferase; ALT: alanine-aminotransferase; T-bil: total bilirubin; BUN: blood urea nitrogen; Cre: creatinine; CRP: C-reactive protein; WBC: white blood cells; Hb: hemoglobin; Plt: platelet; FT_3_: free triiodothyronine; FT_4_: free thyroxine; TSH: thyroid-stimulating hormone; TSAb: thyroid stimulating antibody; TRAb: TSH receptor antibody; GH: growth hormone; ACTH: adrenocorticotropic hormone; IRI: immunoreactive insulin; BE: base excess.

## References

[1] Burch HB, Wartofsky L (1993). Life-threatening thyrotoxicosis: thyroid storm. *Endocrinology and Metabolism Clinics of North America*.

[2] Akamizu T, Satoh T, Isozaki O (2012). Diagnostic criteria and clinic-epideminological features of thyroid strom based on a nationwide survey. *Thyroid*.

[3] Izumi K, Kondo S, Okada T (2009). A case of atypical thyroid storm with hypoglycemia and lactic acidosis. *Endocrine Journal*.

[4] Kobayashi C, Sasaki H, Kosuge K (2005). Severe starvation hypoglycemia and congestive heart failure induced by thyroid crisis, with accidentally induced severe liver dysfunction and disseminated intravascular coagulation. *Internal Medicine*.

[5] Homma M, Shimizu S, Ogata M, Yamada Y, Saito T, Yamamoto T (1999). Hypoglycemic coma masquerading thyrotoxic storm. *Internal Medicine*.

[6] Service FJ (1995). Hypoglycemic disorders. *The New England Journal of Medicine*.

